# A plant growth-promoting bacteria *Priestia megaterium* JR48 induces plant resistance to the crucifer black rot *via* a salicylic acid-dependent signaling pathway

**DOI:** 10.3389/fpls.2022.1046181

**Published:** 2022-11-10

**Authors:** Qi Li, Zhaoqi Hou, Dongqin Zhou, Mingyun Jia, Shipeng Lu, Jinping Yu

**Affiliations:** Jiangsu Key Laboratory for the Research and Utilization of Plant Resources, Institute of Botany, Jiangsu Province and Chinese Academy of Sciences (Nanjing Botanical Garden Mem. Sun Yat-Sen), Nanjing, China

**Keywords:** JR48, plant resistance, crucifer black rot, Xcc, salicylic acid

## Abstract

*Xanthomonas campestris* pv. *campestris* (*Xcc*)-induced black rot is one of the most serious diseases in cruciferous plants. Using beneficial microbes to control this disease is promising. In our preliminary work, we isolated a bacterial strain (JR48) from a vegetable field. Here, we confirmed the plant-growth-promoting (PGP) effects of JR48 *in planta*, and identified JR48 as a *Priestia megaterium* strain. We found that JR48 was able to induce plant resistance to *Xcc* and prime plant defense responses including hydrogen peroxide (H_2_O_2_) accumulation and callose deposition with elevated expression of defense-related genes. Further, JR48 promoted lignin biosynthesis and raised accumulation of frees salicylic acid (SA) as well as expression of *pathogenesis-related* (*PR*) genes. Finally, we confirmed that JR48-induced plant resistance and defense responses requires SA signaling pathway. Together, our results revealed that JR48 promotes plant growth and induces plant resistance to the crucifer black rot probably through reinforcing SA accumulation and response, highlighting its potential as a novel biocontrol agent in the future.

## Introduction

Beneficial microbes are frequent in nature and improve plant growth or help plant to overcome biotic or abiotic stress. Among them, a category of bacteria which colonize in the rhizosphere, referred to as plant growth-promoting rhizobacteria (PGPR), is extensively investigated (Lugtenberg and Kamilova, 2009). Generally, PGPR improve the growth of plants through different approaches, including nitrogen fixation, solubilization of phosphate and potassium, production of phytohormones, secretion of ACC (1-Aminocyclopropane-1-carboxylate) deaminase, etc (Rosier et al., 2018). Except for plant growth-promoting (PGP) characteristics, many beneficial microbes can be utilized as biocontrol agents to control plant diseases, which is a promising strategy as an alternative to chemical control (Fravel, 2005). So far, various biocontrol agents have been discovered and the corresponding mechanisms are distinguished. First, direct competition for nutrients and niches in the rhizosphere among microbes was widely reported (Kamilova et al., 2005). For example, bacteria which produced high concentrations of siderophore effectively inhibited the soil borne pathogen *Ralstonia solanacearum* (Gu et al., 2020). Second, bacteria can produce antibiotics, bacteriocins, antifungal proteins, and volatile antimicrobial compounds to inhibit even kill pathogens. The *Bacillus cereus* strain D13 produced multiple volatile compounds including 3,5,5-trimethylhexanol and decyl alcohol to inhibit the growth of the bacterial blight pathogen *Xanthomonas oryzae* pv. *oryzae* (*Xoo*) (Xie et al., 2018). In addition, induced systemic resistance (ISR) that microbes prime the whole plant for enhanced defense against pathogens or insect herbivores, has been discovered and investigated for decades (Pieterse et al., 2014). For example, the PGP bacterium *Azospirillum brasilense* REC3 could provide systemic protection against *Colletotrichum acutatum* to strawberry plants (Tortora et al., 2012).

*Xanthomonas campestris* pv. *campestris* (*Xcc*) is the notorious bacterial pathogen responsible for causing crucifer black rot disease in *Brassica* species, leading to severe economic losses (Boulanger et al., 2014). *Arabidopsis thaliana* is also a natural host for pathogenic *Xcc*, making the *Arabidopsis*-*Xcc* system a useful model for the study of molecular plant pathology (Rong et al., 2010). As a vascular pathogen, *Xcc* invades its host mainly through natural pores or wound on the plant surface, and spreads along the vascular bundle, resulting in the typical disease symptoms including V-shaped necrotic lesions on leaves and darkening of vascular tissues (Meyer et al., 2005). Ideally, the most effective way to manage plant disease is to develop resistant cultivars in the fields. However, the vague genetic background of crucifer crops and the deficient knowledge of molecular mechanism for resistance against *Xcc* stagnate the development of resistance breeding (Liu et al., 2019). Currently, fungicides are still the most used means to control crucifer black rot disease. Nevertheless, long-term and excessive use of chemicals brought increased drug resistance of pathogens and threatened safeties of ecosystem and food. Hence, using beneficial microbes to implement biocontrol against crucifer black rot is emerging (Fravel, 2005). For example, bacteria isolates from rhizosphere soil of *Brassica campestris* were screened against *Xcc*, and two isolates are effective in black rot management (Mishra and Arora, 2012). Recently, a *Burkholderia anthina* strain HN-8 exhibiting superb degradation activity of diffusible signal factor (DSF) can significantly reduce the severity of black rot disease in radishes and Chinese cabbage (Ye et al., 2020). However, microbial antagonists that control crucifer black rot disease are rarely, and mechanisms of action urgently need to be uncovered.

Plant deploy two layers of immune system to counteract pathogen infection, one is pattern-triggered immunity (PTI) initiated by perception of the conserved pathogen signatures called microbe-associated molecular patterns (MAMPs), the other is effector-triggered immunity (ETI) activated by recognition of pathogen effectors (Jones and Dangl, 2006). Activation of PTI lead to transient calcium influxes, reactive oxygen species (ROS) burst, and mitogen-activated protein kinase (MAPK) cascades, while these responses are often delayed and prolonged in ETI (Zhou and Zhang, 2020). Plant also produce hormones including salicylic acid (SA), jasmonate (JA), and ethylene (ET) to modulate defense response, among which SA plays a crucial regulatory role in resistance against biotrophic and hemi-biotrophic pathogens (Shigenaga et al., 2017). SA is required for both local and systemic acquired resistance, and plants which that are defective in SA biosynthesis always exhibit enhanced susceptibility to pathogens (Wildermuth et al., 2001). To date, few studies reported the role of SA in plant resistance to *Xcc*. For example, expression of *NahG* in *Arabidopsis* effectively prevents free SA accumulation, and loss of accumulation of SA in *NahG* transgenic lines results in higher levels of *Xcc* bacterial growth (O'Donnell et al., 2003). Furthermore, exogenous application of SA leads to enhanced disease resistance to *Xcc* in the moderate resistant cultivar (CR-Hagwang) of kimchi cabbage (O'Donnell et al., 2003).

In our preliminary work, we isolated 92 bacterial strains in April 2019 from the rhizospheric soil samples of a vegetable field in Jurong, Jiangsu, China. Among them, JR48 exhibited prominent PGP potentials (unpublished data). In this study, we first confirmed that JR48 could promote plant growth of *Arabidopsis*, Chinese cabbage, and tomato. Through phylogenetic analysis and morphological observations, we identified JR48 as a *Priestia megaterium* strain. We found that JR48 induced plant resistance to *Xcc* and primed hydrogen peroxide (H_2_O_2_) accumulation and callose deposition. Furthermore, JR48 promoted lignin biosynthesis and enhanced accumulation of frees SA as well as expression of SA-induced genes. We then proved that JR48-induced plant resistance and defense responses was SA-dependent by using SA signaling-defective genotypes. Finally, we demonstrated that JR48 could induce plant resistance to black rot disease in Chinese cabbage and elevate activities of defense-related enzymes. Our study uncovers a novel biocontrol agent for crucifer black rot and its underlying resistance-inducing mechanisms.

## Materials and methods

### Cultivation and identification of bacteria

Strain JR48 was grown on solid nutrient agar (NA) or liquid nutrient broth (NB) medium at 28°C, and stored at -80°C in 30% (v/v) glycerol. To identify JR48, total genomic DNA was extracted using the Tiangen™ Bacterial Genomic DNA kit according to manufacturer’s instructions. *Gyrase subunit B* (*gyrB*) and *16S rRNA* genes were amplified using universal primers (**Table S1**). PCR products were sequenced and the obtained sequences (**Table S2**) were searched on the GenBank database (NCBI) using Basic Local Alignment Search Tool (BLAST) to find the closest matches.

### Detection of nitrogen fixation, phosphate and potassium solubilization

The nitrogen fixation ability of isolated bacteria was tested on Ashby’s N-free medium (containing per liter: 10 g glucose, 0.2 g KH_2_PO_4_, 0.2 g MgSO_4_.7H_2_O, 0.2 g NaCl, 0.1 g CaSO_4_.2H_2_O, 5 g CaCO_3_, and 20 g agar) by streaking cultivation (Aeron et al., 2015). Phosphate-solubilizing bacteria were screened by inoculation on Pikovskaya’s agar medium (containing per liter: 0.5 g yeast extract, 10 g glucose, 5 g Ca_3_(PO_4_)_2_, 0.5 g (NH_4_)_2_SO_4_, 0.2 g KCl, 0.1 g MgSO_4_.7H_2_O, 0.0001 g MnSO_4_.H_2_O, 0.0001 g FeSO_4_.7H_2_O, and 15 g agar). The plates were incubated at 28°C, and a clear halo around the colonies revealed the phosphate had been solubilized by bacteria. Potassium-solubilization ability of bacterial strains was detected by spotting the log phase cultures on Aleksandrov’s agar plates which contain potassium aluminum silicate as sole source of insoluble inorganic potassium. The plates were incubated at 28°C, and were observed for a clearing zone around the colonies (Kumar et al., 2012). All the plates were checked and recorded after incubation for 5 days.

### Determination of ACC deaminase and IAA production

Production of ACC deaminase was confirmed by conducting the following procedures described before (Kumar et al., 2012). First, log phase culture of each strain was harvested and centrifuged at 10,000 g to get cell pellets. Second, the pellets were washed twice with sterile saline and spotted on minimal medium plates containing ACC as sole nitrogen source. Plates were incubated at 28°C for 72 h. Finally, the grown isolates were inoculated in minimal medium with/without ACC, and better growth of tested bacteria with ACC indicates the production of ACC deaminase.

To determine production of IAA, bacterial isolates were grown in Luria Bertani (LB) medium supplemented with 100 mg L^-1^ L-tryptophan and incubated at 28°C for 24 h on a shaker. Exponentially grown culture (1×10^8^ CFU mL^−1^) was centrifuged at 12,000 g for 15 min, and the supernatant was collected and mixed with equal volume of Salkowski’s reagent (12 g L^−1^ FeCl_3_ in 7.9 mol L^–1^ H_2_SO_4_). The mixture was kept in dark place for 30 min and the absorbance was recorded at 530 nm. The concentration of IAA produced per mL of bacteria suspensions was estimated by comparing absorbance with a standard curve (Verma et al., 2017). Briefly, various concentrations of IAA (0, 2, 5, 10, 50 and 200 μg mL^-1^) were prepared in LB medium and then each concentration was mixed with equal volume of Salkowski’s reagent. The absorbance was recorded at 530 nm after incubation for 30 min and the standard curve was obtained by plotting absorbance against the IAA concentration.

### Plant growth-promoting test

For treatment of seedlings, seeds of *A. thaliana* wild type Col-0, Chinese cabbage inbred line “Aijiao Huang”, and tomato cultivar Heinz 1706 were sterilized for sprouting, then equal number of seedlings were distributed on filter paper in petri dishes (90 mm). Bacterial cells were collected by centrifugation and were resuspended in sterile water to required concentrations, 3 mL cell suspensions were added in petri dishes to soak the seedlings. Treated petri dishes were sealed by parafilm and transferred to a growth chamber under 25°C day/22°C night temperature with 80% relative humidity. Fresh weight were measured at 5 to 7 days post treatment.

For treatment of plants, seedlings of Col-0 were transferred into plastic pots containing autoclaved vermiculite, and supplied with modified half-strength Hoagland nutrient solution weekly. Seedlings of Aijiao Huang and Heinz 1706 were transferred into plastic pots containing field soil (pH 7.7, 0.87 g kg^-1^ Total N, 6.4 mg kg^-1^ Available P, 75 mg kg^-1^ Available K, 11.7 g kg^-1^ Organic C). Plants were cultivated in a greenhouse at 25°C with 12 h daytime and 22°C with 12 h nighttime under 75% humidity. Bacterial cells were harvested by centrifugation and resuspended in sterile water to required concentrations. Four-week-old plants were treated with 20 mL cell suspensions by pouring on the soil around the roots. Representative plants were photographed and corresponding indexes were measured at 14 days post treatment.

### Phylogenetic analysis

Partial *16S rRNA* and *gyrB* gene sequences of JR48 and close matches were aligned by MEGA version 7.0 software (Kumar et al., 2016) using ClustalW method and phylogenetic trees were constructed using the maximum likelihood method with 100 bootstrap replicates.

### Spore staining

A single colony of JR48 was picked from a freshly cultured NA plate and inoculated into 3 mL of NB medium, and the inoculum was placed in shaking incubators under 28°C. After 24 h, 10 μL of cell suspension was smeared on a glass slide, and the slide was heated by flame until dry. A drop of 5% malachite green stain was dripped to evenly cover the dried bacterial cells and the glass slide was mildly heated by flame for 30 s. Then the slide was rinsed by sterile water for 30 s, and a drop of 0.5% sand yellow counterstain was dripped to evenly cover the dried bacterial cells. After 3 s, the glass slide was rinsed by sterile water and naturally dried for microscopic examination. The spores were green and vegetative cells were red.

### Pathogen inoculation on *Arabidopsis*

For bacteria inoculation, *Pst* DC3000 was grown at 28°C in KB medium (containing per liter: 29 g Proteose Peptone, 1.5 g K_2_HPO4, 0.74 g MgSO4, 8 mL glycerol) containing 50 μg mL^-1^ rifampicin (Rif) over 24 h. *Xcc* 8004 was cultured at 28°C in NYG medium (containing per liter: 5 g tryptone, 3 g yeast extract, 20 g glycerol) containing 50 μg mL^-1^ Rif over 24 h. Bacterial cells were harvested by centrifugation and were resuspended in sterile water, suspensions of DC3000 and 8004 were adjusted to an optical density at 600 nm (OD_600_) of 0.001. Five-week-old healthy *Arabidopsis* were treated with sterile water or JR48 bacteria suspensions (1×10^6^ CFU mL^−1^) 3 days before inoculation. Then the overground spatially separated leaves of *Arabidopsis* were infiltrated with the bacterial suspensions using a syringe without a needle. To determine the bacterial population in the plants, infiltrated leaves were detached at 0 or 3 days post inoculation (dpi) and leaf disks (7 mm diameter) were ground for serial dilutions. Dilutions were uniformly coated on plates and CFUs were counted after cultivation for 48 h.

For *P. capsici* inoculation, LT263 used in the study was maintained routinely on 10% (v/v) vegetable juice (V8) medium at 25°C in the dark. Zoospore suspensions of LT263 were prepared as described (Li et al., 2020b). To infect *Arabidopsis*, 5 μL of zoospore suspensions (100 zoospores in total) were dripped on both side of midrib. Inoculated leaves were maintained at 25°C in the dark with high humidity for 36 h. Inoculated leaves were photographed under UV light and relative quantification of *P. capsici* biomass was performed to evaluate infection severity as described (Wang et al., 2013). For *B. cinerea* inoculation, B05.10 used in this study was grown on potato dextrose agar (PDA) at 25°C in the dark. Fresh mycelial plugs (4 mm diameter) were inoculated on middle of the leaves and inoculated leaves were maintained at 25°C in the dark with high humidity for 24 h. Inoculated leaves were photographed under natural light and lesion area was measured at the indicated time points.

### Dual culture assay

To test the antagonistic activity of JR48 on *Xcc* 8004, mono-colony of *Xcc* 8004 was cultured at 28°C in 5 mL of NB medium over 24 h, bacterial cells were collected and resuspended in fresh NB medium. The pathogen suspension was adjusted to a concentration of OD_600_ = 0.5, and mixed with dissolved NA medium (50°C) at a ratio of 1:9 to make the pathogen-containing plates. On the other hand, bacterial strains were grown in 3 mL of NB medium for 20 h, bacterial cells were harvested and resuspended using NB medium and the suspensions were adjusted to a concentration of OD_600_ = 5. Finally, three drops of 5 μL bacterial suspension were equidistantly inoculated around the center of plate, one drop of 5 μL NB medium was used as the control. Plates were incubated at 28°C in the dark for 1 to 2 days, and strains which produced clear halos revealed their antagonisms to the pathogen. To test the antagonistic activity of JR48 on LT263, the dual culture assay was performed as previously described (Li et al., 2022).

### DAB staining and callose deposition assay

Five-week-old healthy plants were inoculated with *Xcc* 8004, and leaves were detached at 0 h, 12 h, and 24 h post inoculation (hpi). For determination of H_2_O_2_ accumulation, *Arabidopsis* leaves were stained with 1 mg mL^-1^ DAB solution overnight in the dark, and destained by ethanol until the decolorizing liquid was transparent. Then samples were prepared on a glass slide and observed by optical microscope. For measurement of callose deposition, *Arabidopsis* leaves were stained with aniline blue, and visualized under UV light by a fluorescence microscope as described (Glazebrook, 2005). The number of callose deposition was counted using Image J software according to software instructions.

### RNA isolation and qRT-PCR

Total RNA was extracted from *Arabidopsis* leaves by using an RNA-simple Total RNA Kit (Tiangen) according to the manufacturer’s instructions. *Arabidopsis* cDNA was synthesized by using the SuperScriptIII First-Strand Kit (Invitrogen). Quantitative reverse transcription (qRT) PCR was performed on ABI Prism 7500 Fast Real-Time PCR system by using a SYBR Premix Ex Taq Kit (TaKaRa) following manufacturer’s instructions. *EF1α* was used as an internal reference in qRT-PCR. Data were analysed using the 2^−ΔΔCt^ method. Gene-specific primers used for qRT-PCR are listed in **Table S1**.

### Determination of lignin content

Lignin content was measured according to the method described (Tsuda et al., 2013). Briefly, air-dried samples were suspended in 1 mL acetic acid containing 25% (v/v) acetyl bromide, and treated at 70°C for 30 min. After cooling to room temperature, 0.9 mL of 2 M NaOH and 0.1 ml of 7.5 M hydroxylamine hydrochloride were added, and the volume of each sample was made up to 10 mL with acetic acid. Samples were centrifuged at 1,000g for 5 min and the absorbance of the supernatant was measured at 280 nm to determine the lignin content.

### Measurement of free SA

Free SA was detected and quantified by using high performance liquid chromatography (HPLC) as described (Li et al., 2020a). Briefly, leaves were ground in liquid nitrogen and suspended in 90% (v/v) methanol, each sample was added with 100 mg 3-hydroxy benzoic acid in 100% methanol. Samples were filtered and separated on a C18 analytical column using HPLC (Agilent 1260) and detected using fluorescence (excitation wavelength: 305 nm, emission wavelength: 405 nm). SA content was quantified by area integration of the HPLC peaks.

### Pathogen inoculation on Chinese cabbage

For inoculation assay, suspensions of 8004 were adjusted to a concentration of OD_600_ = 0.1, and mixed with Silwet L-77 (0.02%, v/v) before use. Five-week-old healthy plants were treated with sterile water or bacteria suspensions 3 days before, and the leaf edges perpendicular to midrib were clipped by a sterilized scissor, which was dipped into the pathogen suspension before use. The incisions were evenly dipped with the pathogen suspension again, and inoculated plants were covered with plastic caps to keep moist for 24 h. Inoculated leaves were detached 5 days post inoculation, and the leaf tissues near the incision were homogenized to determine the number of *Xcc* 8004 by gradient dilution. Representative leaves as well as disease symptoms were photographed and lesion areas were measured at 8 to 10 days post inoculation.

### Determination of activities of defense-related enzymes

After inoculation by *Xcc* 8004, leaves of Chinese cabbage were detached at 0 h, 6 h, 12 h, 24 h, 48 h, and 72 h post inoculation. Leaf tissues near the incision were homogenized to determine the activities of defense-related enzymes by using PAL and PPO test kits (Nanjing Jiancheng Bioengineering Institute, Nanjing, China) according to the manufacturer’s instructions, respectively.

## Results

### JR48 has a plant growth-promoting activity

**Figure 1:**
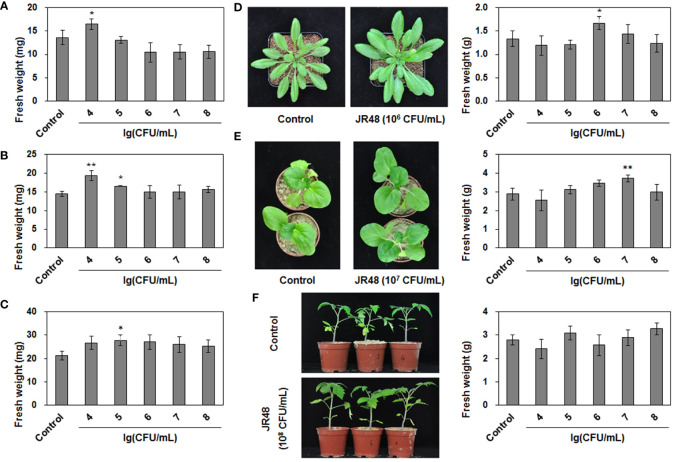
PGP effects of JR48 on plants. **(A-C)** Seedling growth under JR48 treatment. Seedlings of *Arabidopsis* **(A)**, Chinese cabbage **(B)**, and tomato **(C)** were treated with sterile water (control) or indicated bacterial suspensions of JR48 at different concentrations. Fresh weight were measured and calculated at 7 days post treatment. Values are means ± SD (n = 30; *P < 0.05 and **P < 0.01 compared with control, Dunnett’s test). Experiments were repeated in triplicate. **(D-F)** Plant growth under JR48 treatment. *Arabidopsis* **(D)**, Chinese cabbage **(E)**, and tomato **(F)** plants were treated as described above. Representative photographs (left panel) were taken at 14 days post treatment and fresh weight were measured at the same time. Values are means ± SD representative of three independent biological replicates (n = 20; *P < 0.05 and **P < 0.01 compared with control, Dunnett’s test).

In our preliminary work, the bacterial strain JR48 was isolated in a vegetable field. To investigate whether JR48 has potential to promote plant growth, we successively checked its PGP characteristics. As shown in **Table 1**, JR48 could grow in first and second regions on Ashby’s N-free solid medium, indicating a conjectural nitrogen fixation ability. Growth of JR48 resulted in a large clear halo on Pikovskaya’s agar plates and a relatively small halo on Aleksandrov’s agar plates. Furthermore, JR48 could produce ACC deaminase and secreted more than 10 μg mL^-1^ IAA in liquid medium (**Table 1**). Based on this, we tested the PGP effects of JR48 *in planta*. Results showed that 10^4^ CFU/mL of JR48 significantly increased the fresh weight of seedlings of *Arabidopsis* and Chinese cabbage (*Brassica rapa* ssp. *chinensis*) by 21.09% and 33.90% (**Figures 1A, B**), and 10^5^ CFU/mL of JR48 increased the fresh weight of tomato (*Solanum lycopersicum*) seedlings by 30.88% (**Figure 1C**). Furthermore, 10^6^ CFU/mL of JR48 significantly promoted the growth of *Arabidopsis* plants, with a 0.25-fold increase in fresh weight (**Figure 1D**), and 10^7^ CFU/mL of JR48 notably promoted the plant growth of Chinese cabbage, with a 0.29-fold increase in fresh weight (**Figure 1E**). Finally, JR48-treated tomato plants at 10^8^ CFU/mL showed an increase in fresh weight of 17.29% relative to that of control (**Figure 1F**). Together, our results indicated that JR48 could promote seedling and plant growth of *Arabidopsis*, Chinese cabbage, and tomato.

**Table 1:** *In vitro* determination for PGP characteristics of JR48.

Isolates	Nitrogen fixation ^a^	Phosphate solubilization ^b^	Potassium solubilization ^b^	ACC deaminase production ^c^	IAA production (μg mL^-1^) ^d^
JR48	++	+++	+	+	11.86 ± 1.75

^a^ - , no growth; + , growth only in first region; ++ , growth in first and second regions; +++ , growth in three regions. ^b^ - , no growth; + , growth with 0~5 mm clearing zones; ++ , growth with 5~10 mm clearing zones; +++ , growth with more than 10 mm clearing zones. ^c^ ACC; 1-Aminocyclopropane-1-carboxylate; −; negative; +; positive. ^d^ IAA; indole-3-acetic acid; valves are means ± SD (n , 3) from three independent experiments.

### JR48 was identified as *Priestia megaterium*

**Figure 2:**
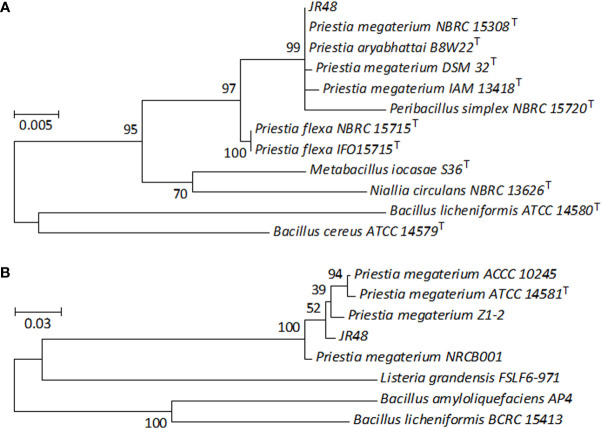
Phylogenetic analysis of JR48. **(A,B)** Phylogenetic tree based on the partial *16S rRNA* gene **(A)** and *gyrB* gene **(B)** sequences of JR48. Phylogenetic tree was constructed by MEGA 7.0 software using the maximum likelihood method with 100 bootstrap replicates. The percentage numbers at the nodes indicate the levels of the associated taxa clustered together. The tree is drawn to scale, with branch lengths were proportional to the number of nucleotide substitutions per site. The scale bar indicates 0.005 **(A)** and 0.03 **(B)** nucleotide substitutions per site. ^T^, type strain.

To identify JR48, we conducted a phylogenetic analysis based on its partial *16S rRNA* gene sequence. The phylogenetic tree showed that the *16S rRNA* gene sequence of JR48 was clustered with several type strains of *Priestia*, and JR48 was most likely to be *P. megaterium* or *P. aryabhattai* (**Figure 2A**), which were previously known as *Bacillus megaterium* and *Bacillus aryabhattai* and fell into the Megaterium clade in phylogenetic tree (Li et al., 2014). Next, we also conducted the phylogenetic analysis of the *gyrase subunit B* (*gyrB*) gene, which has previously been used to distinguish the closely related taxa between *Bacillus* strains (Larsen et al., 2014). Results showed that the partial *gyrB* gene sequence of JR48 was close to the *P. megaterium* strain Z1-2, and clustered with other *P. megaterium* strains including a type strain ATCC 14581 (**Figure 2B**). The partial *16S rRNA* gene and *gyrB* gene sequences of FX2 were deposited in the GenBank database with accession numbers (ON627838 and ON713418). Further, colonies of JR48 on NA plates were slightly raised and light yellow-white, exhibited a slightly shiny surface under visual observation (**Supplementary Figure 1A**). We also found JR48 was rod-shaped, rounded at the end, single or arranged in short chains by using optical microscope. And a few bacterial cells of JR48 formed green spores after cultivation for 24 h (**Supplementary Figure 1B**). Together, all these observations met the morphological characteristics of *P. megaterium*. Thus, JR48 was identified as a *P. megaterium* strain.

### JR48 induces plants resistance to diseases

**Figure 3:**
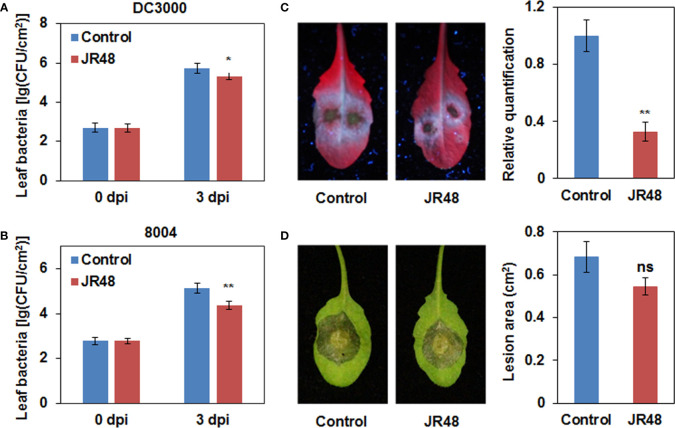
Enhanced plant resistance by JR48. **(A,B)** Bacterial population in *Arabidopsis* leaves. *Pst* DC3000 **(A)** and *Xcc* 8004 **(B)** were infiltrated into leaves of wild-type *Arabidopsis* Col-0 pre-treated with sterile water (control) or JR48. Bacterial population was measured at the indicated time points (mean ± SD; n (number of leaves) ≥ 6; *P < 0.05 and **P < 0.01, Student’s *t*-test). Experiments were repeated in triplicate. **(C)** *P. capsici* inoculation on *Arabidopsis*. Zoospore suspensions of LT263 were dripped on leaves of Col-0 pre-treated with control or JR48. Infected leaves were photographed under UV light. The relative biomass of *P. capsici* was measured by qRT-PCR. Data presented are means ± SD (n ≥ 16), ** indicates significant differences (P < 0.01, Student’s *t*-test). This experiment was repeated at least three times with similar results. **(D)** *B. cinerea* inoculation on *Arabidopsis*. Mycelial plugs of B05.10 were inoculated on leaves of Col-0 pre-treated with control or JR48. Inoculated leaves were photographed under natural light at and lesion area was measured. Values are presented as means ± SD (n ≥ 16) calculated from three independent biological replicates (ns, not significant, Student’s *t*-test).

Previous studies have documented that some beneficial microbes can induce resistance to disease in many plant species (Walters et al., 2013). To test whether JR48 can induce resistance, we conducted inoculation assay on *Arabidopsis* plants pre-treated with sterile water or JR48. *Arabidopsis* is susceptible to the hemi-biotrophic bacterial pathogen *Pseudomonas syringae* pathovar *tomato* (*Pst*) DC3000. Results showed that JR48-treated plants supported less bacterial growth of DC3000 than control, whose bacterial population was 39.72% of that in the control group (**Figure 3A**). JR48-treated plants also significantly supported less bacterial growth of *Xcc* 8004 than control, reduced the bacterial population by 82.94% (**Figure 3B**). *Phytophthora capsici* infects a large number of vegetable crops and the model plant *Arabidopsis* thaliana (Lamour et al., 2012). **Figure 3C** showed that JR48 treatment notably reduced the *P. capsici* infection on *Arabidopsis*, the relative biomass of *P. capsici* in JR48-treatd leaves was 32.8% of that in the control group. Furthermore, JR48 treatment had relatively weak effects on the *Botryis cinerea* infection on *Arabidopsis* (**Figure 3D**), which is a necrotrophic fungus causing gray mold disease on many fruit and vegetable crops. Together, JR48 can induce plant resistance to the above pathogens, and the inhibition of *Xcc* infection was prominent. In addition, JR48 showed no direct inhibitory effects against *Xcc* 8004 in the dual culture assay (**Supplementary Figure 2A**), compared with *Bacillus amyloliquefaciens* FX2 (Li et al., 2022), which produced a clear halo on the plate as a positive control. Furthermore, JR48 also had no inhibitory activities against LT263 (**Supplementary Figure 2B**), compared with *Bacillus licheniformis* LH4-2 (unpublished data), which strongly inhibited the mycelial growth of LT263.

### JR48 primes plant defense responses and affects expression of PTI-related genes

**Figure 4:**
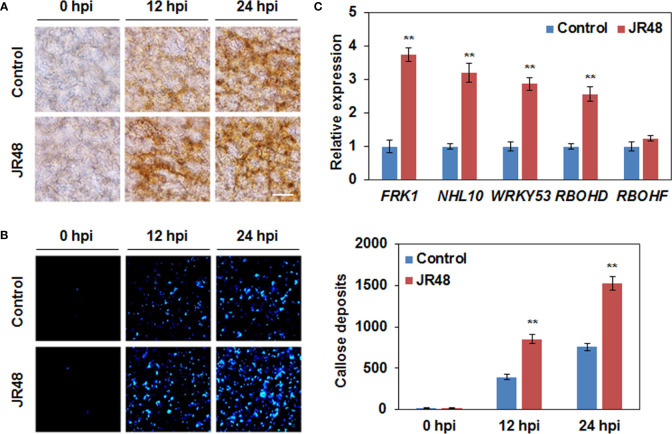
Enhanced plant defense responses and expression of defense-related genes by JR48. **(A)** H_2_O_2_ accumulation during pathogen infection. Leaves of pre-treated wild-type *Arabidopsis* Col-0 were infiltrated with *Xcc* 8004. DAB staining was performed at the indicated time points. Scale bar, 60 μm. **(B)** Callose deposition during pathogen infection. Pre-treated *Arabidopsis* leaves were inoculated with *Xcc* 8004. Leaves were detached for callose staining at the indicated time points. Representative images were photographed and numbers of callose deposition were calculated (mean ± SD; n (number of leaves) ≥ 8). Student’s *t*-test was used to determine significant differences between Control and JR48 (**P < 0.01). Experiments were repeated in triplicate. **(C)** Relative transcript levels of PTI marker genes and two *RBOHs*. Relative expression of the indicated genes in *Arabidopsis* at 12 h post *Xcc* infection was determined by qRT-PCR. *EF1α* was used as an internal reference. Each value represents the mean of three replicates and error bars indicate SD. Asterisks indicate significant differences (**P < 0.01, Student’s *t*-test). This experiment was repeated three times with similar results.

ROS have a direct antimicrobial effect against pathogens, and they also act as local and systemic signal molecules to activate other plant immune response (Ribeiro and Cardoso, 2012; Li et al., 2019). Hydrogen peroxide (H_2_O_2_) is a representative extracellular ROS. Results showed that H_2_O_2_ accumulation was preliminarily observed at 12 hpi in leaves that inoculated with *Xcc* 8004 alone, but JR48-treated together with *Xcc* 8004-inoculated leaves showed darker staining (**Figure 4A**). Moreover, both of them showed similar magnitudes of DAB staining at 24 hpi (**Figure 4A**). Callose deposition is a well-known ROS-initiated defense response for fortification of cell walls (Jones and Dangl, 2006). As shown in **Figure 4B**, significantly stronger signals of callose were detected in JR48-treated together with *Xcc* 8004-inoculated leaves at 12 hpi and 24 hpi. JR48 treatment resulted in 1.17-fold and 1.02-fold increases in number of callose deposition at 12 hpi and 24 hpi (**Figure 4B**). In addition, neither of them showed detectable H_2_O_2_ accumulation and callose deposition at 0 hpi, demonstrating that they were activated by pathogen infection instead of JR48 pre-treatment. ROS production and callose deposition are considered as two typical immune responses in PTI (Chuang et al., 2022). To further clarify whether JR48 can enhance PTI responses, we detect the transcript levels of PTI-related genes using qRT-PCR. Results showed that the relative expression of three PTI marker genes, *FRK1*, *NHL10*, and *WRKY53*, exhibited a 2.73-fold, 2.2-fold, and 1.87-fold increase under JR48 treatment during pathogen infection, compared with those in the control group (**Figure 4C**). We also evaluated the expression levels of two genes encoding respiratory burst oxidase homologues (RBOHs), *RBOHD* and *RBOHF*, which produce ROS to perform a wide range of functions (Lin et al., 2022). As shown in **Figure 4C**, JR48 treatment led to a 1.56-fold increase in the expression level of *RBOHD* during pathogen infection, but had little effect on expression of *RBOHF*, compared with that treated with control. Together, our findings revealed that JR48 could prime H_2_O_2_ accumulation and callose deposition, and activate expression of defense-related genes.

### JR48 promotes lignin biosynthesis and reinforces SA accumulation and response

**Figure 5:**
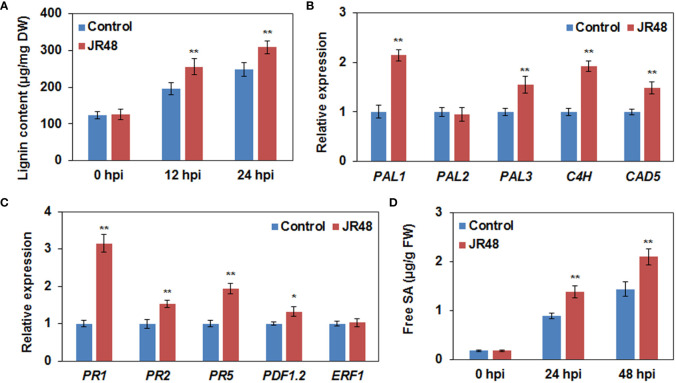
Promoted lignin biosynthesis and reinforced SA accumulation and response by JR48. **(A)** Lignin content during pathogen infection. Leaves of pre-treated wild-type *Arabidopsis* Col-0 were infiltrated with *Xcc* 8004. Lignin content was determined at the indicated time points (mean ± SD; n (number of leaves) ≥ 12; **P < 0.01, Student’s *t*-test). Experiments were repeated in triplicate. DW, dried weight. **(B,C)** Relative expression of genes involved in lignin biosynthesis **(B)** and plant hormone signaling pathway **(C)**. Relative expression of the indicated genes in *Arabidopsis* Col-0 at 12 h post *Xcc* infection was determined. Each value represented is mean of three replicates and error bars indicate SD. Asterisks indicate significant differences (*P < 0.05 and **P < 0.01, Student’s *t*-test). This experiment was repeated three times with similar results. **(D)** Free SA levels during pathogen infection. Pre-treated *Arabidopsis* leaves were inoculated with *Xcc* 8004. Free SA levels were measured at the indicated time points. Data are presented as means ± SD (n ≥ 12) calculated from three independent biological replicates (**P < 0.01, Student’s *t*-test). FW, fresh weight.

Lignin, one of the most abundant cell wall components, plays an important role in cell wall-based defense by building a physical barrier (Ji et al., 2015). Results showed that JR48 treatment increased lignin content in *Xcc* 8004-inoculated plants by 30.14% and 24.21% at 12 hpi and 24 hpi, compared with that treated with control (**Figure 5A**). We then selected some representive genes involved in lignin biosynthesis including three *PALs*, *C4H*, and *CAD5* (Rong et al., 2010), to check their transcript levels. As shown in **Figure 5B**, the relative expression levels of *PAL1*, *PAL3*, *C4H*, and *CAD5* were increased by 1.14-fold, 0.55-fold, 0.92-fold, and 0.49-fold under JR48 treatment during *Xcc* infection, respectively. Except for *PAL2*, whose expression levels were unaffected by JR48 treatment (**Figure 5B**). SA, JA, and ET are considered as crucial plant hormones in regulating plant resistance to biotrophic and necrotrophic pathogens (Glazebrook, 2005). We further determined the expression levels of three SA-induced genes encoding pathogenesis-related (PR) proteins. Results showed that the relative expression of *PR1*, *PR2*, and *PR5*, were significantly increased by 2.15-fold, 0.53-fold, and 0.94-fold increase under JR48 treatment, compared with those in the control group (**Figure 5C**). But the expression levels of two JA/ET-responsive genes, *PDF1.2* and *ERF*, the former was slightly promoted and the latter was unaffected by JR48 treatment (**Figure 5C**). Based on this, we hypothesized that JR48 may strengthen SA signaling. Indeed, JR48 treatment markedly raised the accumulation levels of frees SA during *Xcc* infection by 53.98% and 46.19% at 12 hpi and 24 hpi, compared with that treated with control (**Figure 5D**). Together, these results suggested that JR48 could promote lignin biosynthesis and reinforce SA accumulation and response.

### JR48-induced plant resistance and defense responses requires SA signaling pathway

**Figure 6:**
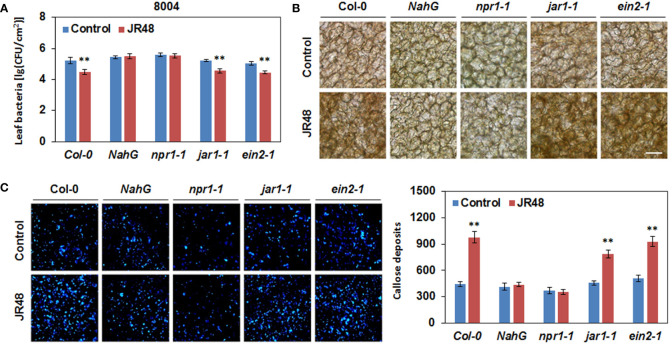
JR48-induced plant resistance and defense responses in Arabidopsis of different genotypes. **(A)** Bacterial growth assay on *Arabidopsis* leaves. Suspensions of *Xcc* 8004 were infiltrated into leaves of Col-0, *NahG*, *npr1-1*, *jar1-1*, and *ein2-1* plants pre-treated with sterile water (control) or JR48. Bacterial population was measured at 3 dpi. Values are presented as mean ± SD (n (number of leaves) ≥ 6) representative of three independent biological replicates. Student’s *t*-test was used to determine significant differences between Control and JR48 (**P < 0.01). **(B)** H_2_O_2_ accumulation during pathogen infection. Leaves of pre-treated *Arabidopsis* Col-0, *NahG*, *npr1-1*, *jar1-1*, and *ein2-1* plants were infiltrated with *Xcc* 8004. DAB staining was performed at 12 hpi. Scale bar, 60 μm. **(C)** Callose deposition during pathogen infection. Pre-treated *Arabidopsis* of different genotypes were inoculated with *Xcc* 8004. Leaves were detached for callose staining at 12 hpi. Representative images were photographed and numbers of callose deposition were calculated (mean ± SD; n (number of leaves) ≥ 8; **P < 0.01, Student’s *t*-test). This experiment was repeated three times with similar results.

To investigate whether JR48-induced plant resistance depends on SA signaling pathway, we conducted bacterial growth assay in *Arabisopsis* of different genotypes. Among them, *NahG* transgenic plants express a bacterial SA hydroxylase and *npr1-1* is a loss-of-function mutant in SA signaling (Ye et al., 2020). In addition, two mutants involved in JA and ET signaling, *jar1-1* (*jasmonate*-*resistant 1-1*) and *ein2-1* (*ethylene-insensitive 2-1*) were also tested (Wildermuth et al., 2001; Zhou and Zhang, 2020). Results showed that JR48-treated Col-0 plants significantly supported less bacterial growth of 8004 than control, but the inactivation of SA signaling in *NahG* and *npr1-1* counteracted JR48-induced resistance, exhibiting similar bacterial numbers of 8004 (**Figure 6A**). In *jar1-1* and *ein2-1*, JR48 treatments also resulted in less bacterial growth of 8004 than control, whose bacterial populations were 22.39% and 25.70% of that in the control group (**Figure 6A**). Similarly, H_2_O_2_ accumulation was preliminarily observed at 12 hpi in Col-0, *jar1-1*, and *ein2-1* in control group, and JR48 treatment led to a darker DAB staining of same magnitudes (**Figure 6B**). In *NahG* and *npr1-1*, JR48 failed to prime H_2_O_2_ accumulation (**Figure 6B**). Furthermore, significantly stronger signals of callose were detected in Col-0, *jar1-1*, and *ein2-1* with JR48 treatment at 12 hpi, numbers of callose deposition were increased by 1.19-fold, 0.72-fold, and 0.81-fold compared with control (**Figure 6C**). But in *NahG* and *npr1-1*, callose deposition were unaffected by JR48 (**Figure 6C**). Thus, JR48 induces plant resistance and defense responses through a SA-dependent signaling pathway.

### JR48 induces plant resistance of Chinese cabbage to black rot disease

**Figure 7:**
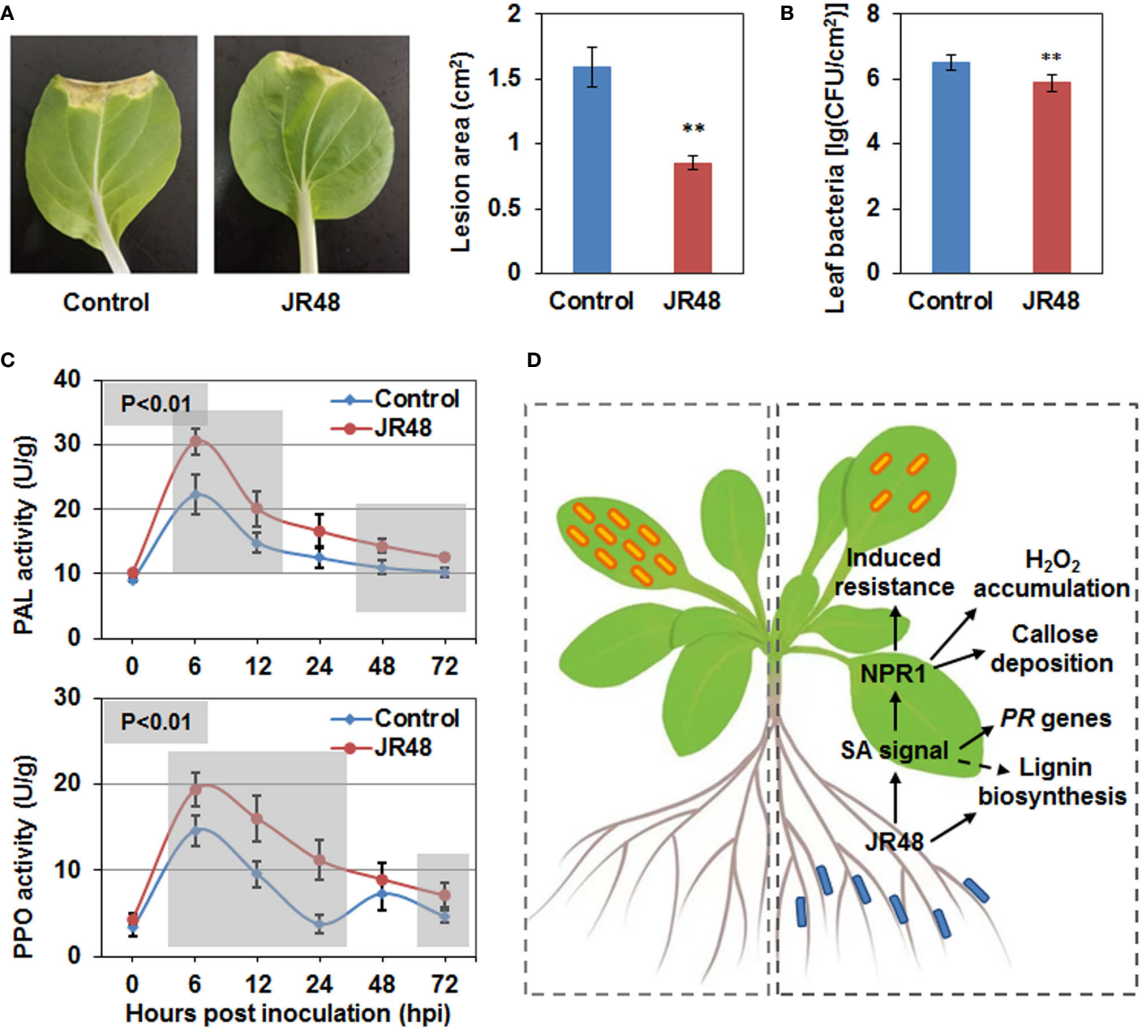
Enhanced plant resistance by JR48 on Chinese cabbage. **(A, B)** Disease symptoms and bacterial infection on Chinese cabbage. Suspensions of *Xcc* 8004 were inoculated on leaves of pre-treated Chinese cabbage by clipping. Representative photographs **(A)** were taken at 10 days post inoculation, lesion area were measured at the same time. Data presented are means ± SD (n ≥ 16), ** indicates significant differences (P < 0.01, Student’s *t*-test). Populations of leaf bacteria **(B)** were counted at 5 days post inoculation (mean ± SD; n ≥ 6; **P < 0.01, Student’s *t*-test). This experiment was repeated three times with similar results. **(C)** Activities of defense-related enzymes during pathogen infection. Chinese cabbage plants were pre-treated and inoculated with *Xcc* 8004 after 3 days. Leaf tissues near the incision were homogenized to determine the activities of PAL and PPO at the indicated time points. The values are means ± SD (n ≥ 12) and significant differences (**P < 0.01) were assessed with Student’s *t*-test. Experiments were repeated in triplicate. **(D)** A schematic diagram illustrating that JR48 induces plant resistance and defense responses to the crucifer black rot *via* a SA-dependent signaling pathway. Left dotted box indicates normal *Xcc* infection on *Arabidopsis*, and small orange rectangles with rounded corners represent *Xcc*. Right dotted box indicates reduced *Xcc* infection on *Arabidopsis* under JR48 treatment, and small blue rectangles with rounded corners represent JR48. One-way solid arrows indicate direct signal transduction and promotion demonstrated in this study, and a dotted arrow indicates indirect promotion.

Considering *Xcc*-induced black rot is a notorious disease in *Brassica* species, we investigated whether JR48 can induce plant resistance in Chinese cabbage (*Brassica rapa* ssp. *chinensis*), which is the most widely grown green leafy vegetable in Asia (Liu et al., 2019). As shown in **Figure 7A**, a typical V-shaped necrotic lesion developed from the incision in the control leaf, while JR48-treated leaves showed smaller and inconsecutive necrotic lesions. JR48 treatment notably reduced the development of necrotic lesions, with a 46.38% decrease in lesion area (**Figure 7A**). Furthermore, JR48-treated plants supported less bacteria populations of *Xcc* 8004 than control plants, the bacteria populations reduced by 76.66%, indicating an effective restriction on pathogen proliferation and infection by JR48 (**Figure 7B**). To further investigate the plant resistance-inducing activity by JR48, we successively assessed activities of two defense-related enzymes. Phenylalanine ammonia lyase (PAL) plays a key role in metabolic pathway of phenyl propane, and manipulates the synthesis of a series of antibiotic compounds (Sumayo et al., 2014). We found PAL activity was induced by pathogen infection and peaked at 6 hpi in control plants, JR48 promoted PAL activity throughout the early stage of infection with the similar pattern (**Figure 7C**). Polyphenol oxidase (PPO) can catalyze the oxidation of phenols to quinones, and involves in synthesis of xylogen. Results showed that PPO activity was also induced by pathogen infection and peaked at 6 hpi and 48 hpi in control plants, JR48 promoted and stabilized PPO activity with one peak value in the early stage of infection (**Figure 7C**). Taken together, JR48 can also induce Chinese cabbage resistance to black rot disease with elevated activities of defense-related enzymes.

## Discussion

Some beneficial microbes have both plant growth-promoting and resistance-inducing abilities. For example, some endophytic bacteria isolated from seeds of *Leersia oryzoides* were capable of increasing root and shoot growth, stimulating root hair formation, and protecting rice seedlings from the soil borne pathogen *Fusarium oxysporum* (Verma et al., 2017). In our study, JR48 had positive effects on seedling and plant growth of *Arabidopsis*, Chinese cabbage, and tomato. Then we validated whether it could induce plant resistance to pathogens. In the non-contact inoculation assay, pre-treatment by JR48 notably reduced the infection of *Pst* DC3000, *Xcc* 8004, and *P. capsici in planta*, while had relatively weak effects on inhibition of *B. cinerea* infection. Considering they are all hemi-biotrophic pathogens except for *B. cinerea*, a typical necrotrophic phytopathogen, these results suggested JR48-induced plant resistance mainly acts on the hemi-biotrophic pathogens. It was widely accepted that SA is a key plant hormone that is required for local and systemic resistance to the early infection stages of hemi-biotrophic pathogens (Glazebrook, 2005). We then hypothesized that maybe JR48 strengthen SA signaling to reduce pathogen infection. Consistently, JR48 significantly promoted expression of *PR* genes, and inactivation of SA signaling in *NahG* and *npr1-1* counteracted JR48-induced resistance (**Figure 7D**). Besides, JR48 had no direct inhibitory effects against *Xcc* 8004 and LT263 in the dual culture assay, demonstrating JR48 could indeed induce plant resistance to the hemi-biotrophic pathogens.

ROS production and callose deposition are two hallmarks of plant early defense response, and often initiated upon MAMP recognition (Jones and Dangl, 2006). Some beneficial microbes are reported to have the ability of activating PTI-associated defense responses. For example, PGPR *Bacillus cereus* AR156 primes for H_2_O_2_ accumulation and callose deposition in *Arabidopsis* during DC3000 infection (Niu et al., 2011). More recently, *Bacillus amyloliquefaciens* PMB05 enhances disease resistance to bacterial wilt caused by *Ralstonia solanacearum*, through initiation of ROS generation and further activation of the MAPK pathway (Chuang et al., 2022). In our study, we found that pre-treatment by JR48 could prime H_2_O_2_ accumulation and callose deposition during pathogen infection, and relative expression of three PTI marker genes and *RBOHD* are also enhanced by JR48. Elevated expression of *RBOHD* was consistent with the DAB staining results, as *RBOHD* is a crucial gene in charge of cellular H_2_O_2_ accumulation in response to MAMP perception (Tang et al., 2019; Lin et al., 2022). In general, these result indicated that JR48 refueled the plant early defense responses by activating PTI signaling. During PTI, SA signaling is required for the proper regulation of the vast majority of SA-responsive genes, ultimately contributes to a major portion of the plant immune response (Tsuda et al., 2013). Based on this, whether SA participated in JR48-induced defense responses was explored. Through using SA signaling-defective genotypes, we found that H_2_O_2_ accumulation and callose deposition induced by JR48 required SA signaling pathway (**Figure 7D**).

Lignin has multiple roles in plant defense, it acts as physical barrier to pathogen invasion and is also required for reinforcing vascular cells (Ji et al., 2015). Lignin biosynthesis by the phenylpropanoid pathway is recruited for biotic stresses, and can be utilized by plants to upon pathogen attack. For example, overexpression of *CsPrx25* form *Citrus sinensis* enhances cell wall lignification, exhibiting significantly increased resistance to *Xanthomonas citri* subsp. *citri* (*Xcc*) infection (Li et al., 2020a). In our study, JR48 treatment increased lignin content in *Arabidopsis* during *Xcc* 8004 inoculation, and promoted relative expression of several genes involved in lignin biosynthesis. These results were reminiscent of a recent study reported that induction of lignin biosynthesis in *Arabidopsis* and rice plays a critical role in plant resistance to vascular pathogens including *Xoo* and *Xcc* (Lin et al., 2022). We also found that JR48 treatment markedly raised accumulation levels of frees SA and increased expression levels of SA-responsive *PR* genes. Similarly, silencing of three homologous cotton *Walls Are Thin* (*WAT*) genes increases plant resistance to verticillium wilt caused by another vascular pathogen, *Verticillium dahlia*, with upregulated expression of SA related genes as well as its biosynthesis and lignin deposition (Tang et al., 2019). PAL catalyze the first step in the phenylpropanoid pathway regulate biosynthesis of lignin and secondary metabolites such as flavonoids and salicylic acid (Ji et al., 2015). Consistent with this, treatment of JR48 promoted PAL activity in the early stage of pathogen infection. Up to now, whether JR48-induced lignification requires SA signaling pathway (**Figure 7D**) and the significance of PAL activity in lignin biosynthesis and SA signaling have not been investigated. Further studies are still needed to explore the underlying resistance-inducing mechanisms by JR48.

In summary, we identified a *P. megaterium* strain JR48 possessing both plant growth-promoting and resistance-inducing abilities. JR48 promoted lignin biosynthesis and reinforced SA accumulation and response. Moreover, JR48-induced plant resistance and defense responses requires SA signaling pathway. This study will advance our understanding of the resistance-inducing mechanisms by a novel biocontrol agent.

## Data availability statement

The datasets presented in this study can be found in online repositories. The names of the repository/repositories and accession number(s) can be found in the article/**Supplementary Material**.

## Author contributions

QL and JY contributed to the study conception and design. Material preparation and experiment implementation were performed by QL, ZH, DZ, MJ, and SL. QL and JY analyzed the data and wrote the manuscript. All authors contributed to the article and approved the submitted version.

## Funding

This work was supported by grants from the National Natural Science Foundation of China (No. 32001959), the Natural Science Foundation of Jiangsu Province (No. BK20200286), the Open Fund of Jiangsu Key Laboratory for the Research and Utilization of Plant Resources (No. JSPKLB202025) and the Special Fund on Technology Innovation of Carbon Dioxide Peaking and Carbon Neutrality of Jiangsu Province (No. BE2022306). The funders had no role in study design, data collection and analysis, decision to publish, or preparation of the manuscript.

## Acknowledgments

We thank Prof. Daolong Dou (Department of Plant Pathology, Nanjing Agricultural University) for providing pertinent suggestions during manuscript preparation.

## Conflict of interest

The authors declare that the research was conducted in the absence of any commercial or financial relationships that could be construed as a potential conflict of interest.

The handling editor declared a shared affiliation with the authors at the time of review.

## Publisher’s note

All claims expressed in this article are solely those of the authors and do not necessarily represent those of their affiliated organizations, or those of the publisher, the editors and the reviewers. Any product that may be evaluated in this article, or claim that may be made by its manufacturer, is not guaranteed or endorsed by the publisher.
