# Supplementary material for: A plant growth-promoting bacteria Priestia megaterium JR48 induces plant resistance to the crucifer black rot via a salicylic acid-dependent signaling pathway
Source: Front Plant Sci. 2022 Nov 10;13:1046181. doi: 10.3389/fpls.2022.1046181 (PMC9684715; doi:10.3389/fpls.2022.1046181)
Supplement: Supplementary file 1 [file DataSheet_1.docx]

**
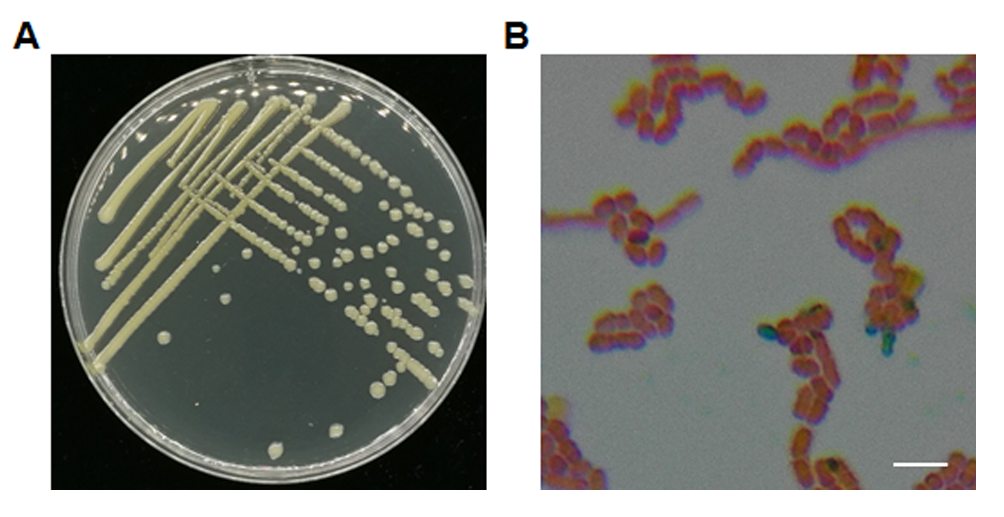
**

**Supplementary Figure 1. Visual observation and spore staining of JR48.**

(A) Visual observation of JR48. JR48 was streaked on a NA plate and cultured at 28°C until single colonies appeared. (B) Spore staining of JR48. Cell suspensions of JR48 were stained by malachite green and counterstained by sand yellow. Green cells indicated spores and red ones indicated vegetative cells. Scale bar, 5 μm.

**
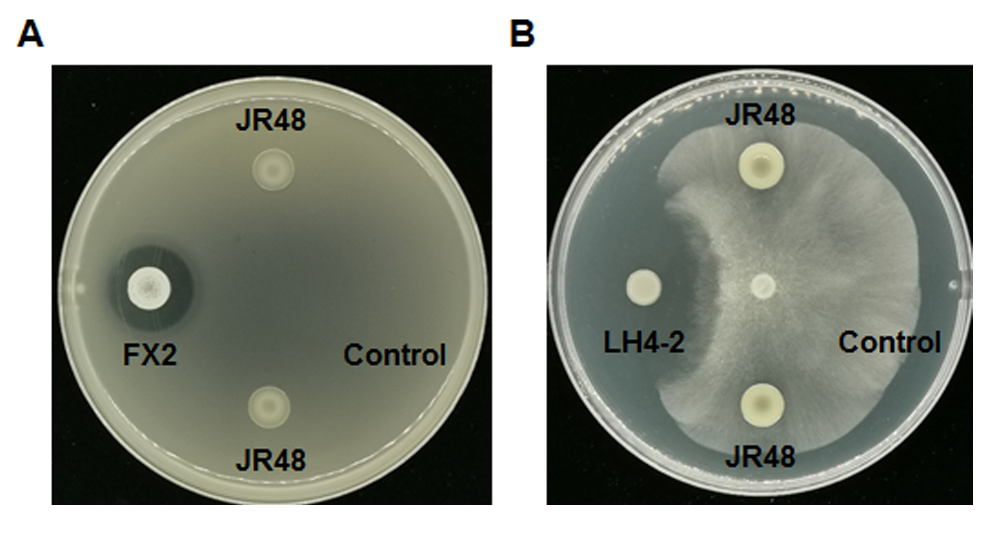
**

**Supplementary Figure 2. Antagonisms of bacterial strains to *Xcc* 8004 and LT263.**

(A) Antagonistic activities on *Xcc* 8004. Pathogen-containing plates were prepared and bacterial suspension were equidistantly dripped on plates. Plates were incubated at 28°C for 1 to 2 days. Control, NB medium; FX2, a positive control. (B) Antagonistic activities on LT263. V8 plates were inoculated with LT263, and bacterial suspension were equidistantly spotted around the pathogen. Plates were incubated at 25°C for 3 to 4 days. Control, NB medium; LH4-2, a positive control.

**Supplementary Table 1. Primers used in this study.**

| **Table S1 Primers used in this study** | | | | |
| --- | --- | --- | --- | --- |
|  | Primer name | Primer sequence | Application |  |
| 1 | *16S rRNA*-F | AGAGTTTGATCCTGGCTCAG | gene cloning |  |
| 2 | *16S rRNA*-R | GGTTACCTTGTTACGACTT | gene cloning |  |
| 3 | *gyrB*-F | YGGHTATAAAGTDTCNGGHGG | gene cloning |  |
| 4 | *gyrB*-R | TCNACRTCBGCRTCBGTCATRAT | gene cloning |  |
| 5 | AtRub-F4 | GCAAGTGTTGGGTTCAAAGCTGGTG | relative quantification of *P. capsici* |  |
| 6 | AtRub-R4 | CCAGGTTGAGGAGTTACTCGGAATGCTG | relative quantification of *P. capsici* |  |
| 7 | CAP-Fw | TTTAGTTGGGGGTCTTGTACC | relative quantification of *P. capsici* |  |
| 8 | CAP-Rv1 | CCTCCACAACCAGCAACA | relative quantification of *P. capsici* |  |
| 9 | *EF1α*-F | CAGGCTGATTGTGCTGTTCTTA | qRT-PCR |  |
| 10 | *EF1α*-R | GTTGTATCCGACCTTCTTCAGG | qRT-PCR |  |
| 11 | *FRK1*-F | GCCAACGGAGACATTAGAG | qRT-PCR |  |
| 12 | *FRK1*-R | CCATAACGACCTGACTCATC | qRT-PCR |  |
| 13 | *NHL10*-F | TTCCTGTCCGTAACCCAAAC | qRT-PCR |  |
| 14 | *NHL10*-R | CCCTCGTAGTAGGCATGAGC | qRT-PCR |  |
| 15 | *WRKY53*-F | CACCAGAGTCAAACCAGCCATTAC | qRT-PCR |  |
| 16 | *WRKY53*-R | CTTTACCATCATCAAGCCCATCGG | qRT-PCR |  |
| 17 | *RBOHD*-F | CTCATTGCCATGCTTCAGTC | qRT-PCR |  |
| 18 | *RBOHD*-R | TTCCTGGCATTCCACAGTAG | qRT-PCR |  |
| 19 | *RBOHF*-F | TCACAAATCAACGACGAGAGTT | qRT-PCR |  |
| 20 | *RBOHF*-R | CCCATCTTCATTCTTGTCCA | qRT-PCR |  |
| 21 | *PAL1*-F | AAGATTGGAGCTTTCGAGGA | qRT-PCR |  |
| 22 | *PAL1*-R | TCTGTTCCAAGCTCTTCCCT | qRT-PCR |  |
| 23 | *PAL2*-F | GGCACCGCATTACAAACAG | qRT-PCR |  |
| 24 | *PAL2*-R | CGGAGTATCCTTGGAGAAGAGT | qRT-PCR |  |
| 25 | *PAL3*-F | TCGTCAACCAAACGCAACAG | qRT-PCR |  |
| 26 | *PAL3*-R | AGTCACTACTCGCCTTCACAC | qRT-PCR |  |
| 27 | *C4H*-F | ACTGGCTTCAAGTCGGAGAT | qRT-PCR |  |
| 28 | *C4H*-R | ACACGACGTTTCTCGTTCTG | qRT-PCR |  |
| 29 | *CAD5*-F | TTGGCTGATTCGTTGGATTA | qRT-PCR |  |
| 30 | *CAD5-*R | ATCACTTTCCTCCCAAGCAT | qRT-PCR |  |
| 31 | *PR1*-F | GAAAACTTAGCCTGGGGTAGC | qRT-PCR |  |
| 32 | *PR1*-R | TTCATTAGTATGGCTTCTCGTTCA | qRT-PCR |  |
| 33 | *PR2*-F | GCAATGCAGAACATCGAGAA | qRT-PCR |  |
| 34 | *PR2*-R | TCATCCCTGAACCTTCCTTG | qRT-PCR |  |
| 35 | *PR5*-F | TATCGGCCGGAATAGGCTCTG | qRT-PCR |  |
| 36 | *PR5*-R | CGCGTACATACAAATGCGTGC | qRT-PCR |  |
| 37 | *PDF1.2*-F | CCATCATCACCCTTATCTTCGC | qRT-PCR |  |
| 38 | *PDF1.2*-R | TGTCCCACTTGGCTTCTCG | qRT-PCR |  |
| 39 | *ERF1*-F | TTAATTCAGTCCCCATTCTC | qRT-PCR |  |
| 40 | *ERF1*-R | CCAAGTCCCACTATTTTCAG | qRT-PCR |  |

**Supplementary Table 2. Partial *16S rRNA* and *gyrB* gene sequences of JR48.**

| **Table S2 Partial *16S rRNA* and *gyrB* gene sequences of JR48** | | |
| --- | --- | --- |
|  | target gene | partial sequecne |
| 1 | *16S rRNA* | CGACTTCGGGTGTTACAAACTCTCGTGGTGTGACGGGCGGTGTGTACAAGGCCCGGGAACGTATTCACCGCGGCATGCTGATCCGCGATTACTAGCGATTCCAGCTTCATGTAGGCGAGTTGCAGCCTACAATCCGAACTGAGAATGGTTTTATGGGATTGGCTTGACCTCGCGGTCTTGCAGCCCTTTGTACCATCCATTGTAGCACGTGTGTAGCCCAGGTCATAAGGGGCATGATGATTTGACGTCATCCCCACCTTCCTCCGGTTTGTCACCGGCAGTCACCTTAGAGTGCCCAACTAAATGCTGGCAACTAAGATCAAGGGTTGCGCTCGTTGCGGGACTTAACCCAACATCTCACGACACGAGCTGACGACAACCATGCACCACCTGTCACTCTGTCCCCCGAAGGGGAACGCTCTATCTCTAGAGTTGTCAGAGGATGTCAAGACCTGGTAAGGTTCTTCGCGTTGCTTCGAATTAAACCACATGCTCCACCGCTTGTGCGGGCCCCCGTCAATTCCTTTGAGTTTCAGTCTTGCGACCGTACTCCCCAGGCGGAGTGCTTAATGCGTTAGCTGCAGCACTAAAGGGCGGAAACCCTCTAACACTTAGCACTCATCGTTTACGGCGTGGACTACCAGGGTATCTAATCCTGTTTGCTCCCCACGCTTTCGCGCCTCAGCGTCAGTTACAGACCAAAAAGCCGCCTTCGCCACTGGTGTTCCTCCACATCTCTACGCATTTCACCGCTACACGTGGAATTCCGCTTTTCTCTTCTGCACTCAAGTTCCCCAGTTTCCAATGACCCTCCACGGTTGAGCCGTGGGCTTTCACATCAGACTTAAGAAACCGCCTGCGCGCGCTTTACGCCCAATAATTCCGGATAACGCTTGCCACCTACGTATTACCGCGGCTGCTGGCACGTAGTTAGCCGTGGCTTTCTGGTTAGGTACCGTCAAGGTACAAGCAGTTACTCTTGTACTTGTTCTTCCCTAACAACAGAGTTTTACGACCCGAAAGCCTTCATCACTCACGCGGCGTTGCTCCGTCAGACTTTCGTCCATTGCGGAAGATTCCCTACTGCTGCCTCCCGTAGGAGTCTGGGCCGTGTCTCAGTCCCAGTGTGGCCGATCACCCTCTCAGGTCGGCTATGCATCGTTGCCTTGGTGAGCCGTTACCTCACCAACTAGCTAATGCACCGCGGGCCCATCTGTAAGTGATAGCCGAAACCATCTTTCAATCATCTCCCATGAAGGAGAAGATCCTATCCGGTATTAGCTTCGGTTTCCCGAAGTTATCCCAGTCTTACAGGCAGGTTGCCCACGTGTTACTCACCCGTCCGCCGCTAACGTCATAGAAGCAAGCTTCTAATCAGTTCGCTCGACTTGC |
| 2 | *gyrB* | CCAGTCCCGAGTGACTCGTTGTTACGCGCTTTCTACCTCTTTGGAAGTGCACGTCCATCGTGACGGTAAAGTTCATTATCAAAAATATGAACGAGGTGTACCGGCTGCTGACTTAAAAGTAGTTGGAGAAACAGATAAAACAGGTACTGTTATTCAATTCCATCCAGACGGTGAAATTTTTACAGAAACGCTTGAATACGATTTTGATACGTTAGCTAATCGTCTGCGTGAGTTAGCTTTCTTAAATCGCGGTATTAAAATTACGATTGAAGACAAACGTGAAGAAGACAAAAGACGTGAATATCATTATGAAGGCGGAATTAAGTCTTACGTTGAACACTTAAACCGTTCGAAAGAAGTGATTCACGAAGAGCCGATCTATATTGAAGGTAATCGAGACAACATTTCTGTAGAAATTGCTATTCAATATAACGATAGCTATACAAGTAATTTATATTCTTTTGCAAACAACATTCACACATATGAAGGTGGAACGCACGAAGCAGGATTTAAAACAGCGTTAACGCGTGTAATTAACGACTATGCACGTAAAAATAGCGTATTTAAAGACAGTGACGCCAATCTAACGGGTGAAGATGTTCGTGAAGGAATTACAGCTATCATCTCTATTAAGCACCCAGATCCGCAGTTCGAAGGACAAACAAAAACAAAGCTGGGAAATAGTGAAGCAAGAACAATTACTGATTCTGTGTTTGCAGAACACTTAGAAACTTACTTGCTAGAGAACCCTATTGTGGCGAAAAAGGTAATTGAAAAAGGTTTAATGGCTGCAAGAGCAAGAATGGCAGCTAAAAAAGCGCGTGAGCTTACAAGACGTAAAAGCGCGCTTGAAATTTCAAACTTACCGGGTAAATTAGCAGATTGTTCATCAAAAGATCCTTCTATTAGCGAACTCTATGTAGTAGAGGGTGACTCTGCCGGAGGTTCAGCTAAACAGGGAAGAAGCCGTCATTTCCAAGCTATTTTGCCTTTACGTGGTAAAATTATCAACGTAGAGAAAGCGCGTTTAGATAAAATTTTATCTAATAACGAAATTCGTACAATCATTACCGCTCTAGGAACGGGTATTGGTGACGATTTGATATCGAAGCACGCAGCTTT |
